# Expectations for papers performing Mendelian randomization analyses

**DOI:** 10.1371/journal.pgen.1011767

**Published:** 2025-07-17

**Authors:** Scott M. Williams, Hua Tang, Gregory M. Cooper, Anne O’Donnell-Luria, Santhosh Girirajan, Aimée M. Dudley, Anne Goriely, Zoltán Kutalik, Xiaofeng Zhu, Giorgio Sirugo, Michael P. Epstein

**Affiliations:** 1 Department of Population and Quantitative Health Sciences, Case Western Reserve University School of Medicine, Cleveland, Ohio, United States of America; 2 Department of Genetics, Stanford University School of Medicine, Stanford, California, United States of America; 3 HudsonAlpha Institute for Biotechnology, Huntsville, Alabama, United States of America; 4 Program in Medical and Population Genetics, Broad Institute of MIT and Harvard University, Cambridge, Massachusetts, United States of America; 5 Division of Genetics and Genomics, Boston Children’s Hospital, Boston, Massachusetts, United States of America; 6 Department of Biochemistry and Molecular Biology, The Pennsylvania State University, University Park, Pennsylvania, United States of America; 7 Pacific Northwest Research Institute, Seattle, Washington, United States of America; 8 Radcliffe Department of Medicine, University of Oxford, Oxford, United Kingdom; 9 Department of Epidemiology and Health Systems, Center for Primary Care and Public Health, Unisanté, University of Lausanne, Lausanne, Switzerland; 10 Division of Translational Medicine and Human Genetics, University of Pennsylvania School of Medicine, Philadelphia, Pennsylvania, United States of America; 11 Department of Human Genetics, Emory University School of Medicine, Atlanta, Georgia, United States of America

Mendelian randomization (MR) is an analytical construct that was developed with the intent of using genetic segregation to reduce confounding from observational epidemiological studies and hence improve our ability to infer causation between some modifiable risk factors and outcomes [1,2]. It uses the natural process of meiosis that randomly assorts alleles, similar to the process of treatment assignment in a clinical trial. As with clinical trials, reverse causality is only a minor concern for genetic analysis, making MR a potent means to assess causative pathways. The potential of MR to infer causation has led to a surge of papers using it as an analytical approach. The incredible growth of MR studies has also been supported by the development of numerous software packages that can perform MR analyses. Some of these can even perform multiple MR analyses with ease (e.g., Yavorska and Burgess [3]). Additionally, the availability of publicly available genome-wide association summary statistics for a wide range of phenotypes has further fueled the growth of MR research. Taken together, all these factors appear on their face positive.

However, with the promise of MR and the increasing availability of data and software, several problems have emerged; critical flaws have been identified in many MR studies, including numerous manuscripts submitted to *PLOS Genetics* in recent years. As simply noted in a recent review, successful use of MR to assess the causal role of modifiable risks, as well as association with other disease outcomes, hinges on three key assumptions [4]. First, the genetic variants used as instrumental variables must be strongly associated with the exposure or the potentially modifiable environmental risk factor of interest. The second assumption is that the genetic variants influence the outcome or disease only through the exposure being tested [4]. A third assumption is that the genotype is independent of confounding factors affecting the outcome. As noted in a review of the literature, as of 2015, fewer than half of MR studies adequately explored the validity of these assumptions [5]. We note that submissions to *PLOS Genetics* are consistent with this number. Nonetheless, these assumptions require explicit evaluation, consideration and clear documentation.

Even at its outset, it was recognized that MR as a methodological construct had limitations [1]. We are concerned that the recent upsurge of MR studies often overlooks the concerns raised early in the development of the method. To this point *PLOS Genetics* has received many manuscripts involving poorly performed MR studies that are either unaware of the rigorous methodological care necessary for proper implementation or simply disregard it for convenience of producing more publications. The majority of these MR manuscripts go little beyond the simple automated application of existing standard methods of the standard R packages (such as TwoSampleMR [6], https://mrcieu.github.io/TwoSampleMR/) to publicly available data, generating standard plots and tables with practically no added value. These manuscripts are almost invariably rejected during editorial review. Our goal in this editorial is to remind readers and authors of the initial intents of MR analyses, the conditions necessary for their valid applications, and the inherent limitations of the method. To do this we outline below the specific criteria that MR papers must meet before being considered for publication in *PLOS Genetics*.

A key feature of MR is that the genetic variant(s) used as instrumental variables are strongly associated with the exposure of interest. Therefore, prior knowledge supporting such associations is essential and needs to be explicitly described and annotated in the manuscript. In essence, authors need to specify what is the explicit relationship being tested and provide the justification for the analyses. Sensitivity analyses testing the robustness of the results upon various instrument selection criteria are essential. Using multiple independent Genome Wide Association Studies (GWAS) to estimate the effect sizes also increases robustness. As originally proposed, MR was best used as a means of assessing causation but its utility for this depends on the extent to which the key assumptions can be validated. If used as a discovery tool, multiple testing correction and careful consideration of the underlying scope of prior probabilities for the hypotheses being tested need to be performed. That said, we consider such discovery studies to be of low priority, unless accompanied by follow-up analyses that seek complementary evidence of support.

Next, researchers need to be aware of the assumptions of the specific method or software package being used and how sensitive the results are to violations of these assumptions. For example, multiple statistical approaches have been developed to detect violations of MR assumptions or to relax some assumptions under defined conditions. Comparing results across these approaches (many of which are outlined and compared in Hu and colleagues [7]) can provide insight into the robustness and credibility of the findings.

In a similar vein, it is critical to assess whether the assumptions of MR have been explicitly tested. Explicit testing of instrument pleiotropy via pheWAS, and the application and comparison of pleiotropy robust MR methods of complementary assumptions is a must, as horizontal pleiotropy directly threatens the validity of causal inference and remains inadequately addressed in most MR studies currently submitted to *PLOS Genetics*. Multivariable MR methods have recently been developed to alleviate correlated pleiotropy, and analyzing multiple correlated risk factors in MR is encouraged besides univariable MR [8]. In addition, careful analysis of heterogeneity statistics is key to understanding and reducing assumption violations [9]. The more rigorous the testing of assumptions, the more reliable the resulting inferences are. Such considerations are fundamental aspects of a well-executed MR study and more broadly of high-quality, rigorous scientific research that we expect, in general.

Another consideration is whether the results are robust to analyses in multiple populations. Population stratification has long been argued to confound associations between genotype and disease in case-control studies, but it can also be problematic for MR studies [1,10]. Therefore, it is important to consider the populations being analyzed in relation to the populations from which prior genotype-exposure associations were derived, and to assess whether the associations are plausibly transferable. Also, we think that, whenever possible, the generalizability of the MR results should be evaluated and discussed. These assessments should become increasingly possible given the growing availability of diverse datasets. This is not to say that a strong result must be universally transferable, but the breadth of the results needs to be assessed and discussed. Statistical power should be presented given the relatively smaller sample sizes in populations other than Europeans when interpreting results. Explicit instrument selection in different populations should be well justified.

We also expect that findings from MR analysis be supported by complementary lines of evidence, ideally including validation in appropriate experimental models. While experimental testing is not strictly necessary nor always feasible, consistent with our general policy of publishing GWAS results, we strongly encourage authors to provide some sort of biological validation of their MR findings [11]. In addition, negative control experiments can further boost the reliability of potential positive results.

Lastly but importantly, it is critical that the interpretation of the results be appropriate and credible. This means two things. First, that the study truly adds value to our understanding of a key biological process leading to a disease or trait. Second, that the interpretation is consistent with the nature of the phenomena being analyzed and the results observed. This is of course necessary for any paper *PLOS Genetics* elects to publish but given the large number of implausible or ambiguous conclusions observed in manuscript submissions over recent months, this requirement is of particular importance for MR studies and for maintaining the rigor and quality of publications.

## References

[1] Smith GD, Ebrahim S. Mendelian randomization: prospects, potentials, and limitations. Int J Epidemiol. 2004;33(1):30–42. doi: 10.1093/ije/dyh132 . 15075143

[2] Smith GD, Ebrahim S. “Mendelian randomization”: can genetic epidemiology contribute to understanding environmental determinants of disease? Int J Epidemiol. 2003;32(1):1–22. doi: 10.1093/ije/dyg070 . 12689998

[3] Yavorska OO, Burgess S. Mendelian randomization: an R package for performing Mendelian randomization analyses using summarized data. Int J Epidemiol. 2017;46(6):1734–9. doi: 10.1093/ije/dyx034 . 28398548 PMC5510723

[4] Burgess S, Woolf B, Mason AM, Ala-Korpela M, Gill D. Addressing the credibility crisis in Mendelian randomization. BMC Med. 2024;22(1):374. doi: 10.1186/s12916-024-03607-5 . 39256834 PMC11389083

[5] Boef AGC, Dekkers OM, le Cessie S. Mendelian randomization studies: a review of the approaches used and the quality of reporting. Int J Epidemiol. 2015;44(2):496–511. doi: 10.1093/ije/dyv071 . 25953784

[6] Hemani G, Zheng J, Elsworth B, Wade KH, Haberland V, Baird D, et al. The MR-Base platform supports systematic causal inference across the human phenome. Elife. 2018;7:e34408. doi: 10.7554/eLife.34408 . 29846171 PMC5976434

[7] Hu X, Cai M, Xiao J, Wan X, Wang Z, Zhao H, et al. Benchmarking Mendelian randomization methods for causal inference using genome-wide association study summary statistics. Am J Hum Genet. 2024;111(8):1717–35. doi: 10.1016/j.ajhg.2024.06.016 . 39059387 PMC11339627

[8] Sanderson E, Davey Smith G, Windmeijer F, Bowden J. An examination of multivariable Mendelian randomization in the single-sample and two-sample summary data settings. Int J Epidemiol. 2019;48(3):713–27. doi: 10.1093/ije/dyy262 . 30535378 PMC6734942

[9] Bowden J, Del Greco M F, Minelli C, Zhao Q, Lawlor DA, Sheehan NA, et al. Improving the accuracy of two-sample summary-data Mendelian randomization: moving beyond the NOME assumption. Int J Epidemiol. 2019;48(3):728–42. doi: 10.1093/ije/dyy258 . 30561657 PMC6659376

[10] Thomas DC, Witte JS. Point: population stratification: a problem for case-control studies of candidate-gene associations? Cancer Epidemiol Biomarkers Prev. 2002;11(6):505–12. . 12050090

[11] Barsh GS, Copenhaver GP, Gibson G, Williams SM. Guidelines for genome-wide association studies. PLoS Genet. 2012;8(7):e1002812. doi: 10.1371/journal.pgen.1002812 . 22792080 PMC3390399

